# Local Application of Sodium Salicylate Enhances Auditory Responses in the Rat’s Dorsal Cortex of the Inferior Colliculus

**DOI:** 10.3389/fneur.2014.00235

**Published:** 2014-11-14

**Authors:** Chirag R. Patel, Huiming Zhang

**Affiliations:** ^1^Department of Biological Sciences, University of Windsor, Windsor, ON, Canada

**Keywords:** auditory system, GABA_A_ receptor, GABA_B_ receptor, hearing, midbrain, tinnitus, microiontophoresis

## Abstract

Sodium salicylate (SS) is a widely used medication with side effects on hearing. In order to understand these side effects, we recorded sound-driven local-field potentials in a neural structure, the dorsal cortex of the inferior colliculus (ICd). Using a microiontophoretic technique, we applied SS at sites of recording and studied how auditory responses were affected by the drug. Furthermore, we studied how the responses were affected by combined local application of SS and an agonists/antagonist of the type-A or type-B γ-aminobutyric acid receptor (GABA_A_ or GABA_B_ receptor). Results revealed that SS applied alone enhanced auditory responses in the ICd, indicating that the drug had local targets in the structure. Simultaneous application of the drug and a GABAergic receptor antagonist synergistically enhanced amplitudes of responses. The synergistic interaction between SS and a GABA_A_ receptor antagonist had a relatively early start in reference to the onset of acoustic stimulation and the duration of this interaction was independent of sound intensity. The interaction between SS and a GABA_B_ receptor antagonist had a relatively late start, and the duration of this interaction was dependent on sound intensity. Simultaneous application of the drug and a GABAergic receptor agonist produced an effect different from the sum of effects produced by the two drugs released individually. These differences between simultaneous and individual drug applications suggest that SS modified GABAergic inhibition in the ICd. Our results indicate that SS can affect sound-driven activity in the ICd by modulating local GABAergic inhibition.

## Introduction

Sodium salicylate (SS) is a widely used medication with side effects on hearing (1–5). The side effects include temporary hearing loss and transient tinnitus (6–11). In animal models, SS can cause conditioned behaviors as if experimental subjects perceived phantom sounds (12–19).

The effect of SS on hearing is likely related to changes caused by the drug in physiological processes in peripheral and central auditory structures [see Ref. (5, 20–22) for reviews]. In the auditory periphery, the drug can bind onto anion binding sites of the motor protein prestin (23). Such binding suppresses the electromotility of outer hair cells and reduces the output of the sensory organ (24–26). In spite of this reduction in the output, amplitudes of sound-driven local-field potentials (LFPs) in the central auditory structure, the inferior colliculus (IC), are not changed following systemic injection of SS (22, 27). The amplitudes of LFPs in the auditory cortex (AC) and the medial geniculate nucleus are even enhanced by the same drug manipulation (16, 22, 27, 28). These findings suggest that SS can directly change physiological processes in the central auditory system, which compensates for the drug-induced reduction in the output from the auditory periphery. In addition to sound-driven responses, spontaneous firing of neurons in structures such as the AC and the external cortex of the IC (ICx) can also be enhanced by systemic injection of SS (29–31). These results also suggest that the drug can directly affect physiological processes in central auditory structures. Existing results indicate that the effect of SS on auditory neural responses is predominantly restricted to non-lemniscal parts of the auditory system (5, 21, 22).

*In vitro* neurophysiological/pharmacological methods have been used to investigate mechanisms through which SS affects neural activity. Experiments conducted in the AC, IC, hippocampal cornu ammonis area 1 (i.e., CA1 area), and spinal dorsal horn suggest that SS can affect inhibitory neurotransmission mediated by γ-aminobutyric acid (GABA) (32–35).

As the IC is a structure essential for hearing, it is significant to examine the effect of SS on auditory activities in the structure. There is evidence showing that GABAergic inhibition plays a major role in auditory responses in the IC (36–39) and SS can modulate GABAergic neurotransmission in the structure (34). Therefore, it is possible that the drug can change sound-driven responses in the structure through modulating local GABAergic neurotransmission. Area differences exist in the effect of SS on neural activity in the IC. For instance, while systemic injection of the drug leads to an enhancement in spontaneous activity in neurons in the ICx (29), it causes a reduction in spontaneous activity in neurons in a specific area of the central nucleus of the IC (ICc) (40).

The effect of SS on neural activities in the dorsal cortex of the IC (ICd) has not been evaluated so far. As a non-lemniscal part of the auditory midbrain, this structure has high levels of GABA (41–44) and GABAergic receptors (42, 45–50). Therefore, the present study was designed to investigate the effect of local SS on auditory responses in the ICd and to find whether such an effect was related to a change in local GABAergic inhibition. Sound-driven LFPs were recorded in the ICd. SS and a GABAergic receptor agonist/antagonist were released individually as well as simultaneously at sites of recording using a microiontophoretic technique. Our results indicate that local SS can enhance auditory responses in the ICd. Such enhancement is likely related to a modulation of GABAergic inhibition.

## Materials and Methods

### Animal preparation

Experiments were conducted using 26 male adult Wistar albino rats (*Rattus norvegicus*) with body weights between 250 and 500 g. The rats were obtained from Charles River Canada Inc., Saint Constant, QC, Canada. Anesthesia was induced by combined injections of ketamine hydrochloride (60 mg/kg, i.m.) and xylazine hydrochloride (10 mg/kg, i.m.) and was maintained by supplemental injections of ketamine hydrochloride (20 mg/kg, i.m.) and xylazine hydrochloride (3.3 mg/kg, i.m.). Recordings were performed when a rat was inside a CL-15A LP single-wall sound-attenuated booth (Eckel Industries, Morrisburg, ON, Canada). The rat’s head was held firmly by a headbar attached to a Model 900 stereotaxic instrument (Kopf Instruments, Tujunga, CA, USA). A small craniotomy was made for placing an electrode assembly into the left ICd. Procedures were approved by the University of Windsor Animal Care Committee and were in accordance with the guidelines of the Canadian Council on Animal Care.

### Acoustic stimulation

Acoustic waveforms were generated digitally using a System 3 real-time signal processing system controlled by a personal computer running OpenEx software (Tucker-Davis Technologies, Alachua, FL, USA). Sounds were delivered to the ear contralateral to the recording site (i.e., the right ear) using a CF1 closed-field speaker (Tucker-Davis Technologies, Alachua, FL, USA) connected to a short piece of Tygon tubing inserted in the rat’s external meatus. Brief monaural tone bursts with 2-ms rise/fall times (without a plateau) were used to elicit auditory responses. The sound-generating system was calibrated over a frequency range between 100 and 45,000 Hz using a 7017 condenser microphone (ACO pacific, Belmont, CA, USA).

### Electrodes for physiological recordings and pharmacological manipulations

A piggy-back electrode assembly as described in a previous publication (51) was used in experiments. The single-barrel recording electrode (impedance 300–500 kΩ) of the assembly was filled with either 2 M NaCl or 3% *N*-(2-aminoethyl) biotinamide hydrochloride (Neurobiotin, Vector Laboratories SP-1120, Burlingame, CA, USA) in 0.5 M sodium acetate.

Of the five barrels of the drug-releasing pipette, one was filled with SS (50 mM, pH 3.5, Sigma-Aldrich S3007, Oakville, ON, Canada). The second and third barrels were filled with bicuculline methiodide (BMI, 25 mM, pH 3.5, Sigma-Aldrich B6889, Oakville, ON, Canada), an antagonist for the type-A GABAergic receptor (GABA_A_ receptor), and CGP35348 (25 mM, pH 3.5, Sigma-Aldrich C5851, Oakville, ON, Canada), an antagonist for the type-B GABAergic receptor (GABA_B_ receptor). Alternatively, the two barrels were filled with the GABA_A_ receptor agonist muscimol (Musc, 10 mM, pH 3.5, Sigma-Aldrich M1523, Oakville, ON, Canada) and the GABA_B_ receptor agonist baclofen (Bac, 10 mM, pH 3.5, Sigma-Aldrich B5399, Oakville, ON, Canada), respectively. The remaining barrels were filled with 165 mM NaCl. The vehicle of Bac was a citrate buffer, while that of the other drugs was 165 mM NaCl. Drugs were released iontophoretically using a Neurophore BH-2 microiontophoresis system (Harvard Apparatus, Saint Laurent, QC, USA).

### Experimental procedures

An electrode assembly was driven into the left ICd from a dorso-rostro-lateral to a ventro-caudo-medial location of the brain [see Ref. (51, 52) for details] using a model 2660 micropositioner (Kopf, Tujunga, CA, USA). A −20 nA retention current was applied to each barrel with a pharmacological agent when the electrode assembly was in the brain. Neural signals were amplified by a 2400 A preamplifier (Dagan, Minneapolis, MN, USA) and sampled at 24.414 kHz using the System 3 real-time signal processing system.

**Figure 1:**
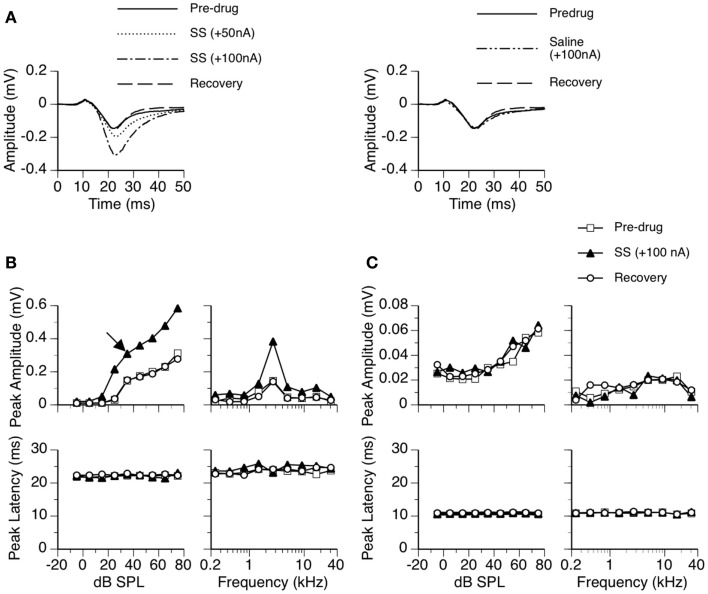
**An example showing the effect of SS on an LFP recorded in the ICd**. **(A)** Left panel: LFPs evoked by a tone burst at the BF (2.7 kHz) and 10 dB above the MT of the recording site [as indicated by an arrow in the top left panel of **(B)**] before, during, and after application of SS. **(A)** Right panel: an LFP evoked by the same sound when a microiontophoretic current of 100 nA (the maximum current used in drug release in this case) was applied to an electrode barrel containing saline vehicle alone. LFPs recorded before application of SS and after recovery are also shown in the panel. **(B,C)** Dependences of the peak amplitude (upper panels) and the peak latency (bottom panels) of the D_N_ **(B)** and the D_P_ **(C)** on the sound-pressure level (left panels) and sound frequency (right panels). In both **(B,C)**, results shown in the left panels were obtained at the BF of the recording site. Results shown in the right panels were obtained at 20 dB above the MT of the recording site.

For each rat, sound-evoked LFPs were recorded at a single site. Trains of contralaterally presented tone bursts were used to search for a site where large LFPs could be elicited. Each train had 9 tone bursts with their frequencies equally spaced on a logarithmic scale. The 9 tone bursts were presented in a randomized order at a constant rate of 4/s and a fixed level of 70 dB SPL. A sound-evoked LFP had a relatively small positive deflection (D_P_) followed by a large negative deflection (D_N_) (see Figure 1A, for example). The total duration of the response was about 40 ms. Upon identification of a recording site, trains of tone bursts with reduced sound-pressure levels and frequency ranges were used to determine the best frequency (BF) and the threshold at BF (i.e., the minimum threshold or MT) at this site.

An LFP in response to a BF tone burst was recorded at the MT and multiple sub- and supra-threshold sound-pressure levels. Peak amplitude by sound-pressure-level functions and peak latency by sound-pressure-level functions were created for the D_P_ and D_N_ of an LFP. A tone burst was presented 300 times at each level for producing an averaged evoked LFP. Tone burst presentations (Total number: number of levels × 300) were produced in a randomized order at a rate of 4/s.

An LFP was also recorded at multiple (typically 9) frequencies at a fixed sound-pressure level at 10–30 dB above the MT. These frequencies were evenly spaced on a logarithmic scale, with the middle frequency equal to the BF and the range covering the frequency response area of the recording site. Peak amplitude by sound-frequency functions and peak latency by sound-frequency functions were created for the D_P_ and D_N_ of an LFP. A tone burst was presented 300 times at each frequency for generating an averaged evoked LFP. Tone burst presentations (Total number: number of frequencies × 300) were produced in a randomized order at a rate of 4/s.

Sodium salicylate and a receptor agonist (Musc or Bac)/antagonist (BMI or CGP35348) were released individually or in combination. Under each drug condition, dependences of peak amplitudes and peak latencies of the D_P_ and D_N_ on sound-pressure level and sound frequency were examined when a drug-induced increase/reduction reached a saturated level with no further changes over a period of at least 15 min. For each case, SS was always the first drug released. After the effect of SS reached a saturated level, a neurotransmitter receptor agonist or antagonist was released while the application of SS was maintained. After the effect of combined drug application reached a saturated level, the release of SS was terminated and the effect of the agonist or antagonist was examined. The release of the agonist or antagonist was then discontinued and recovery was observed while a holding current was re-applied.

In 9 of the 26 rats, LFPs were also recorded when the largest combined current used in drug releases was applied to a barrel containing a vehicle alone. The purpose was to ensure that changes in auditory responses during microiontophoresis of drugs were indeed caused by drugs but not electrical currents. These recordings were conducted after effects of SS and agonists/antagonists were examined and auditory responses fully returned to pre-drug levels. Results from each of the nine cases indicated that microiontophoretic currents did not change the waveform of an LFP. The right panel of Figure 1A shows an example of an LFP recorded when a current was applied to an electrode barrel containing vehicle alone.

In spite of the fact that our stereotaxic coordinates result in consistent placement of an electrode in the ICd (51), the site of recording in the ICd was verified in two randomly chosen rats in the present study. Following physiological recordings, neurobiotin was released using a microiontophoretic current (5 μA, 7-s on/off times, total duration 15–25 min) generated by a Midgard Precision Current Source (Stoelting, Wood Dale, IL, USA). Twenty minutes after the completion of drug release, the rat was given an overdose of sodium pentobarbital (120 mg/kg, i.p.) and transcardially perfused with 4% paraformaldehyde. After the brain was extracted, it was cryoprotected in 30% sucrose for overnight. It was then frozen sectioned at 40 μm in the frontal plane using a CM1050 S cryostat (Leica Microsystems, Heidelberg, Germany). An immunohistological reaction was conducted by using streptavidin-Alexa Fluor 568 conjugate (Molecular Probes, Eugene, OR, USA) and the labeling was examined using a CTR 6500 microscope (Leica Microsystems).

### Data analysis

The effect of a drug/drugs was evaluated by comparing the peak amplitude and latency of a deflection (D_N_ or D_P_) obtained before and during application of the drug/drugs. A difference waveform between the LFPs recorded during and before drug application was used to evaluate the time course of drug-inducted changes.

A change in the amplitude of LFP caused by simultaneous application of SS and a receptor agonist/antagonist was compared with the sum of the changes caused by the two drugs applied individually. A disparity between simultaneous and individual applications in the total effect of the drugs was used to indicate an interaction between the two drugs during simultaneous application. Such interaction would suggest that a modulation of GABAergic pathways/neurotransmission by SS. A larger total effect during simultaneous application would indicate a synergistic interaction between two drugs (53).

Statistical analysis was conducted by using IBM SPSS Statistics 22 and a custom-made script based on MatLab and Simulink (R2008b).

## Results

The effect of SS on sound-driven LFPs was studied in all the 26 rats (named as cases elsewhere in the text) used in this study. In a subgroup of nine cases, we examined effects of BMI applied individually and in combination with SS. In a subgroup of eight cases, we examined effects of CGP35348 applied individually and in combination with SS. There were seven cases used in both the groups. For each of the seven cases, effects of BMI (alone and in combination with SS) were examined first. Effects of CGP35348 (alone and in combination with SS) were examined after an LFP completely recovered from effects of BMI and SS. Effects of Musc and Bac (each applied individually and in combination with SS) were investigated in two separate subgroups with six and seven cases, respectively. Effects of GABAergic receptor agonists/antagonists (alone and in combination with SS) were not tested in the remaining three cases.

### Effects of SS on LFPs in the ICd

Sodium salicylate increased the amplitude of an LFP over the duration of the D_N_ in a current-dependent manner. For the example shown in Figure 1, the peak amplitude was increased by about 50% when SS was released at 50 nA and was more than doubled at 100 nA (Figure 1A left panel). The increase in the peak amplitude of D_N_ was observed over a wide range of intensities and frequencies (Figure 1B top panels). The peak latency of D_N_ was not affected by the drug (Figures 1A,B bottom panels). The D_N_ typically restored its pre-drug shape about 20 min after the cessation of drug release (Figures 1A,B), indicating that the effect of SS was reversible.

**Figure 2:**
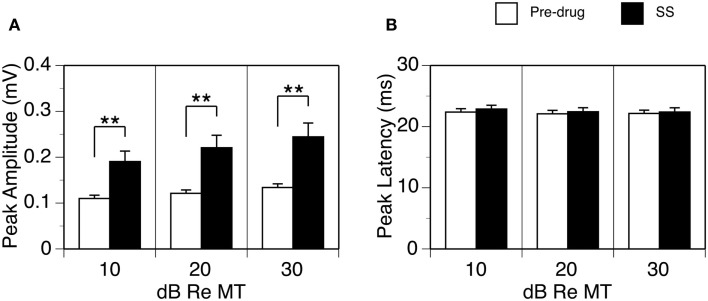
**Group results (*n* = 26) showing effects of SS on the peak amplitude (A) and peak latency of the D_N_ (B)**. For making **(A,B)**, data from each animal were collected at the BF and 10, 20, and 30 dB above the MT of a recording sites. Double stars indicate statistical significance at the level of *p* < 0.001. Error bars represent SE.

The effect of SS on the peak amplitude of an LFP was examined at 10, 20, and 30 dB above the MT in each of the 26 cases. Group results indicated that the effect of SS was significant [Figure 2A; two-way repeated-measures ANOVA, *F*_(1,25)_ = 24.28, *p* < 0.001]. *Post hoc* analysis revealed that the increase caused by the drug was significant at all intensities (Tukey pairwise comparisons with Bonferroni correction, *p* < 0.001 at all intensities). No statistical differences existed between the peak latencies of the D_N_ recorded before and during the drug (Figure 2B).

The waveform of D_P_ including its peak amplitude and latency was not affected by SS (see Figures 1A,C, for example). Thus, no further analyses were conducted on this deflection.

**Figure 3:**
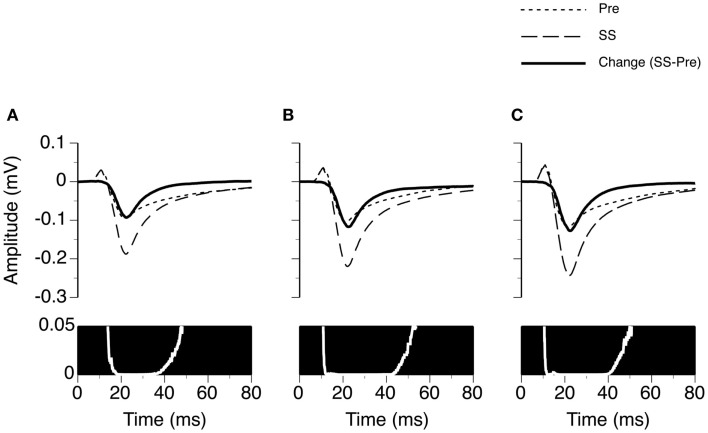
**Group results (*n* = 26) showing the time window over which SS enhances the amplitude of an LFP recorded at the BF of the recording site**. **(A–C)** are based on LFPs recorded at 10, 20, and 30 dB above the MT of a recording site. In the upper panels of **(A–C)**, dotted and dashed lines represent grand-mean waveforms of LFPs recorded before and during SS, respectively. A solid black line is the difference between two grand-mean waveforms. A white line with a black background is a *p*-value (Wilcoxon signed-rank test) by time function comparing the difference between the amplitudes of the two grand-mean waveforms.

We examined the time window over which SS significantly increased the amplitude of an LFP (Figure 3). At each specific stimulus level, two LFPs recorded before and during application of SS were paired for each of the 26 cases. The resulting 26 pairs of LFPs were used to generate two grand-mean waveforms (top panels in Figures 3A–C). Each waveform (either a recorded trace or a grand mean) had 6104 data points, as a recording was conducted over a 250 ms period at a sampling rate of 24.414 kHz. A Wilcoxon signed-rank test was conducted at each of these 6104 points using 26 pairs of amplitude values obtained before and during application of SS. A resulting *p*-value was used to indicate the level of significance of the change caused by SS at this sampling point. A *p*-value by time curve obtained at 10 dB above the MT indicated that SS significantly increased the amplitude of an LFP (*p* < 0.05) at all points over a time window between 13.8 and 48.1 ms after the onset of a stimulus (bottom panel in Figure 3A). This time window was to some extent wider at higher stimulus levels (compare Figures 3B,C bottom panels with the Figure 3A bottom panel).

### Interactions between SS and GABAergic receptor antagonists in shaping LFPs

**Figure 4:**
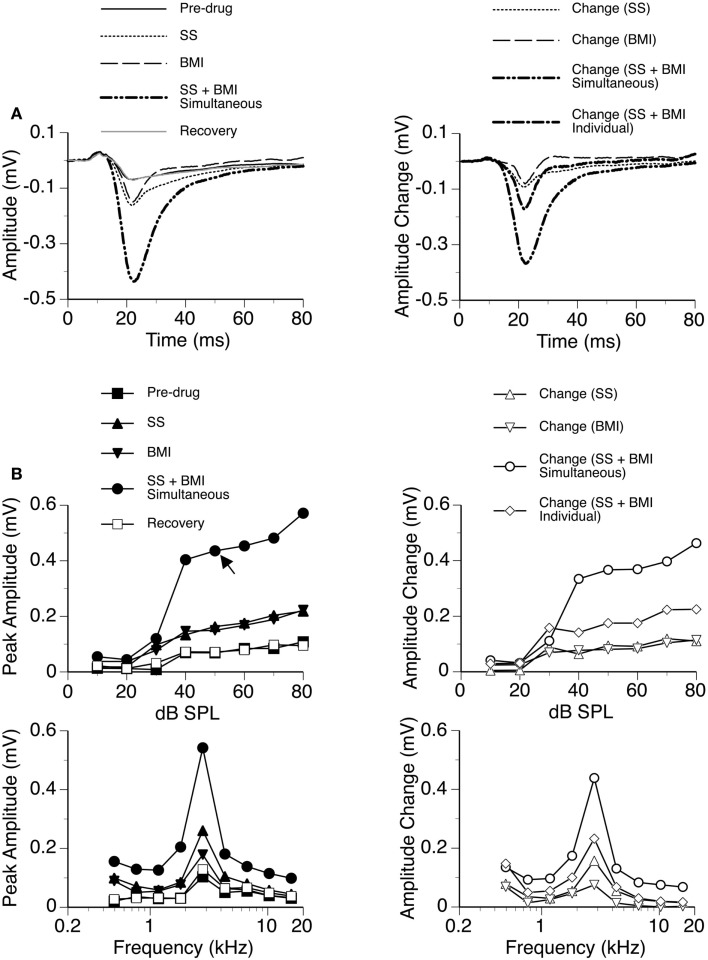
**An example showing the interaction between SS and BMI**. **(A)** Left panel: LFPs evoked by a tone burst at the BF (2.8 kHz) and 20 dB above the MT of a recording site [as indicated by an arrow in the top left panel of **(B)**] before drug, during individual and simultaneous applications of SS and BMI, and after recovery. **(A)** Right panel: difference waveforms showing changes caused by individual and simultaneous applications of SS and BMI. Also, shown in this panel is a waveform resulting from summation of changes caused by individual applications of SS and BMI. **(B)** Left panels: amplitude by sound-pressure level (top) and amplitude by sound-frequency (bottom) functions of the D_N_ obtained before drug, during individual and simultaneous applications of SS and BMI, and after recovery. **(B)** Right panels: changes in the peak amplitude of the D_N_ caused by individual and simultaneous applications of SS and BMI at various sound-pressure levels (top) and sound frequencies (bottom). Also, shown in each of the right panels in **(B)** is a curve resulting from summation of changes caused by individual applications of SS and BMI.

In nine cases, sound-driven LFPs were recorded when SS was released along with the GABA_A_ receptor antagonist BMI. For the example shown in Figure 4, the peak amplitude of the D_N_ at 20 dB above the MT was increased by SS (released at 100 nA) and BMI (released at 20 nA) by about 140 and 120%, respectively (Figure 4A). Simultaneous application of the two drugs resulted in an increase in peak amplitude by more than 530% (Figure 4A), which was much larger than the sum of the increases caused by the two drugs individually (a total of 260%). This disparity suggested that a synergistic interaction existed between SS and BMI in regulating an LFP. Such an interaction was observed over a wide range of intensities and frequencies (Figure 4B). Effects of SS and BMI peaked at almost the same time (Figure 4A). Nevertheless, the effect of SS lasted longer than that of BMI.

**Figure 5:**
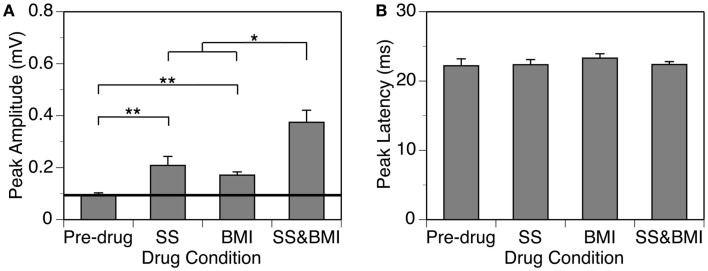
**Group results (*n* = 9) obtained at 10 dB above the MT showing effects of individual applications of SS and BMI (two middle bars) and a simultaneous application of the drugs (the right bar) on the peak amplitude (A) and the peak latency (B) of the D_N_**. The horizontal line in **(A)** represents the mean peak amplitude of the D_N_ obtained before drug application. Double stars indicate statistical significance at the level of *p* < 0.005. A single star indicates statistical significance at the level of *p* < 0.05. Error bars represent SE.

Group results from nine cases (Figure 5A) confirmed findings from the example shown in Figure 4. As results under different drug conditions (i.e., control, SS alone, SS + BMI, and BMI alone) were obtained from the same nine animals over time, a two-way repeated-measures ANOVA was used for analyzing these results. An interaction between the two group factors SS and BMI was examined. At 10 dB above the MT, SS, and BMI released individually caused significant increases in the peak amplitude of the D_N_ [*F*_(1,8)_ = 106.79, *p* < 0.001 for SS; *F*_(1,8)_ = 23.14, *p* < 0.005 for BMI]. The two drugs released simultaneously caused an increase larger than the sum of the increases caused by the two drugs applied individually [*F*_(1,8)_ = 8.631, *p* < 0.05]. Results obtained at other supra-threshold intensities were consistent with those obtained at 10 dB above the MT. Neither individual nor combined application of SS and BMI changed the peak latency of the D_N_ (Figure 5B).

**Figure 6:**
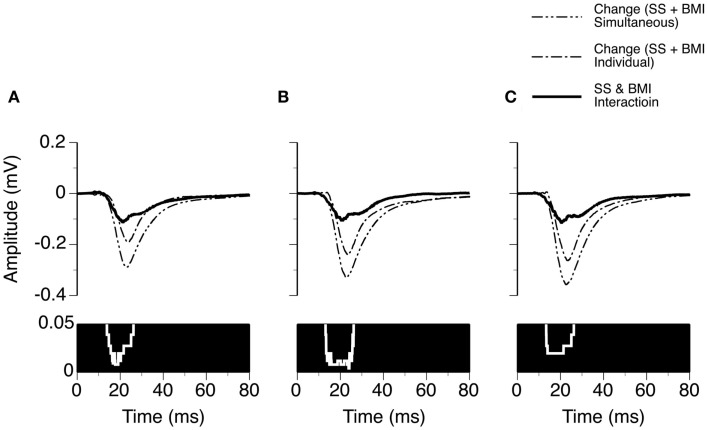
**Group results (*n* = 9) showing time windows over which a synergistic interaction exists between SS and BMI in enhancing the amplitude of an LFP recorded at the BF of a recording site**. **(A–C)** are based on results obtained at 10, 20, and 30 dB above the MT of a recording site. In **(A–C)**, a white line with a black background in the bottom panel is a *p*-value by time function showing statistical significance of the difference between the total changes caused by individual and simultaneous applications of SS and BMI.

We examined the time window over which SS and BMI had a synergistic interaction in regulating LFPs. Two waveforms were formed at each sound-pressure level (10, 20, or 30 dB above the MT) for each case. One was a difference waveform between the LFPs obtained before and during simultaneous application of SS and BMI (reflecting a total increase caused by the drugs applied simultaneously). The other one was the sum of the two difference waveforms resulting from individual applications of the drugs (reflecting a total increase caused by the drugs applied individually). Pairs of these waveforms from nine individual cases were utilized to generate two grand-mean waveforms (Figures 6A–C upper panels). At each of the 6104 sampling points (250 ms recording trace sampled at 24.414 kHz), we examined the disparity between the total increase caused by SS and BMI applied simultaneously and the total increases caused by the drugs applied individually (Wilcoxon signed-rank test, data from nine cases). Such a disparity was used to reflect a synergistic interaction between SS and BMI. At 10 dB above the MT, a disparity (*p* < 0.05) existed at all sampling points within a time window between 13.8 and 26.1 ms after the onset of a stimulus (Figure 6A). Similar time windows of interaction were observed at 20 and 30 dB above the MT (13.1–26.2 and 13.4–26.2 ms after the onset of a stimulus, respectively) (Figures 6B,C).

**Figure 7:**
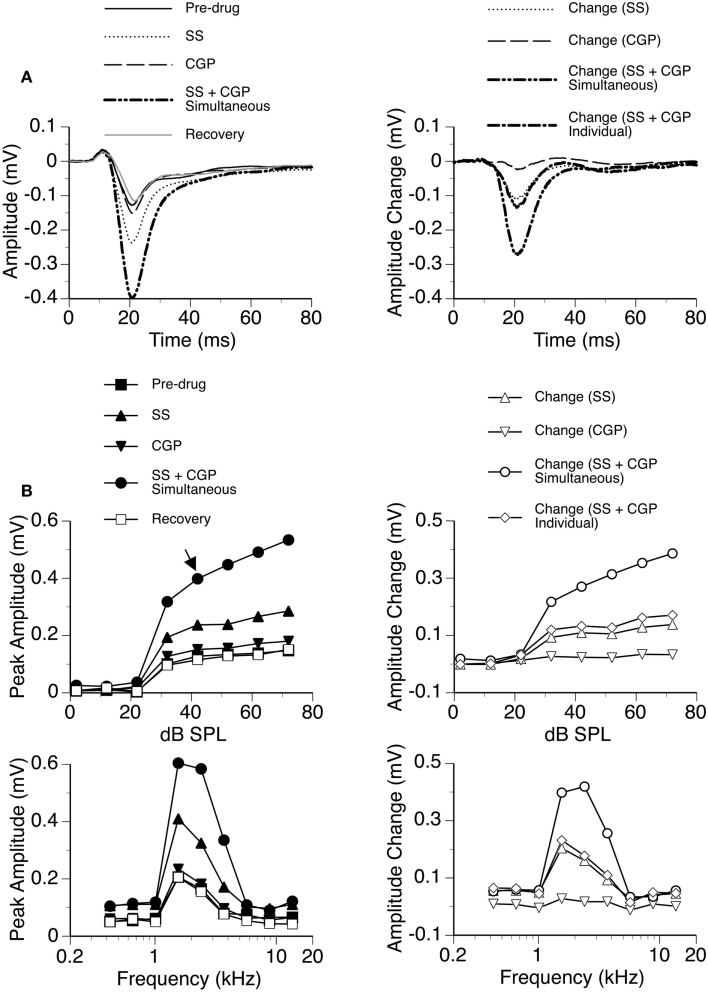
**An example showing the interaction between SS and CGP35348**. **(A)** Left panel: LFPs evoked by a tone burst at the BF (2.4 kHz) and 20 dB above the MT of a recording site [as indicated by an arrow in the top left panel of **(B)**] before drug, during individual and simultaneous applications of SS and CGP35348, and after recovery. **(A)** Right panel: difference waveforms showing changes caused by individual and simultaneous applications of SS and CGP35348. Also, shown in this panel is a waveform resulting from summation of changes caused by individual applications of SS and CGP35348. **(B)** Left panels: amplitude by sound-pressure level (top) and amplitude by sound-frequency (bottom) functions obtained before drug, during individual and simultaneous applications of SS and CGP35348, and after recovery. **(B)** Right panels: changes in the peak amplitude of an LFP caused by individual and simultaneous applications of SS and CGP35348 at various sound-pressure levels (top) and sound frequencies (bottom). Also, shown in each of the right panels of **(B)** is a curve resulting from summation of changes caused by individual applications of the drugs.

In eight cases, recordings were conducted when SS was released along with the GABA_B_ receptor antagonist CGP35348. While SS produced a large increase in the amplitude of the D_N_, the increase caused by CGP35348 was moderate (Figure 7). These effects of the drugs were observed over a wide range of sound intensities and frequencies. The two drugs applied simultaneously produced an increase much larger than the sum of the increases caused by the two drugs applied individually, suggesting that SS and CGP35348 enhanced an LFP synergistically.

**Figure 8:**
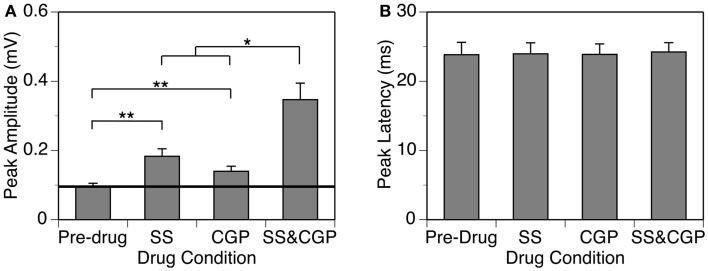
**Group results (*n* = 8) obtained at 10 dB above the MT showing effects of individual applications of SS and CGP35348 (two middle bars) and a simultaneous application of the drugs (right bar) on the peak amplitude (A) and latency (B) of the D_N_**. A horizontal line in **(A)** represent the mean peak amplitude of the D_N_ obtained before drug application. Double stars indicate statistical significance at the level of *p* < 0.005. A single star indicates statistical significance at a level of *p* < 0.05. Error bars represent SE.

Group results from eight cases (Figure 8A) confirmed findings from the example shown in Figure 7. At 10 dB above the MT, individual applications of SS and CGP35348 caused significant increases in the peak amplitude of the D_N_, while simultaneous application of the drugs caused an increase much larger than the sum of those caused by individual applications of the drugs [two-way repeated-measures ANOVA, *F*_(1,7)_ = 49.99, *p* < 0.001 for SS; *F*_(1,7)_ = 22.17, *p* < 0.005 for CGP35348; *F*_(1,7)_ = 6.78, *p* < 0.05 for the interaction between drugs]. Results obtained at other supra-threshold sound-pressure levels were consistent with those obtained at 10 dB above the MT. Group results indicated that SS and CGP35348 did not affect the peak latency of the D_N_ (Figure 8B).

**Figure 9:**
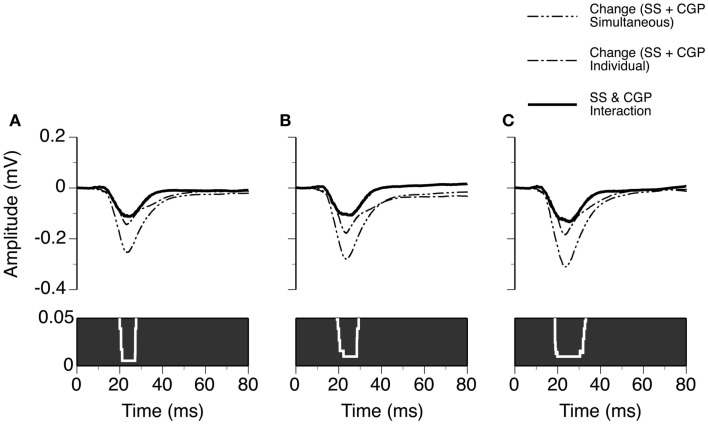
**Group results (*n* = 8) showing the time window over which a synergistic interaction exists between SS and CGP35348 in enhancing the amplitude of an LFP recorded at the BF of a recording site**. **(A–C)** are results obtained at 10, 20, and 30 dB above the MT of a recording site, respectively. In **(A–C)**, a white line with a black background in the bottom panel is a *p*-value by time function showing statistical significance of the difference between the total changes caused by individual and simultaneous applications of SS and CGP35348.

Using results from the eight cases and the same method as used for analyzing results presented in Figure 6, we evaluated the time window over which SS and CGP35348 had a synergistic interaction in regulating the amplitude of an LFP (Figure 9). At 10 dB above the MT, an interaction was observed at each time point over a time window between 20.0 and 27.6 ms after the onset of stimulation (Wilcoxon signed-rank test, *p* < 0.05). This time window of interaction was increased to 19.2–29.3 and 18.8–33.1 ms at 20 and 30 dB above the MT, respectively (Wilcoxon signed-rank test at each data point, *p* < 0.05).

### Interactions between SS and GABAergic receptor agonists in shaping LFPs

**Figure 10:**
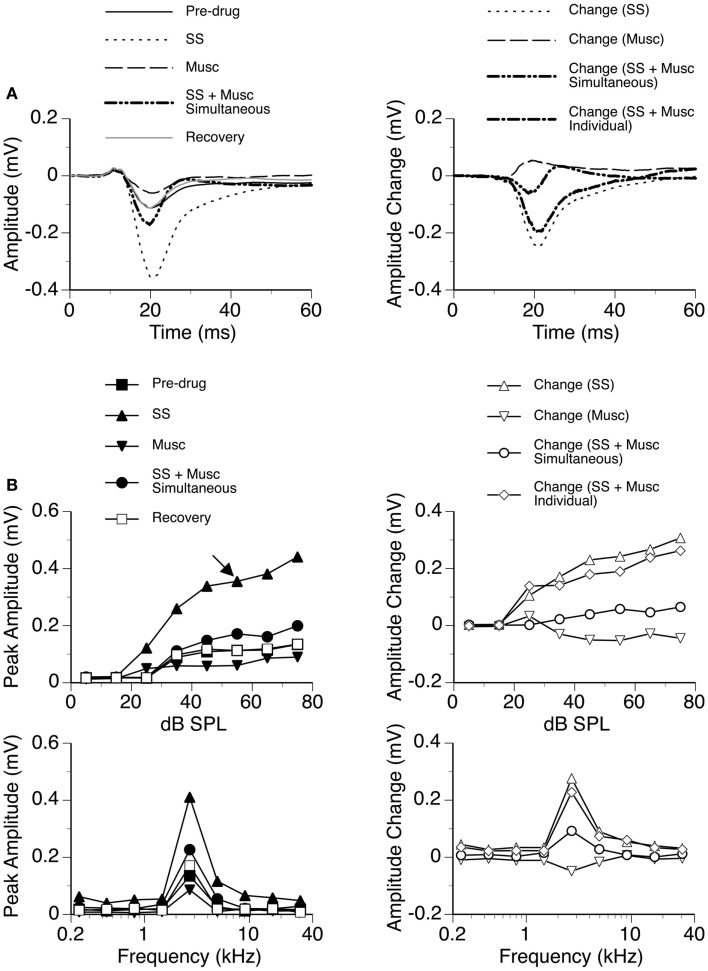
**An example showing the interaction between SS and Musc**. **(A)** Left panel: LFPs evoked by a tone burst at the BF (2.7 kHz) and 30 dB above the MT of the recording site [as indicated by an arrow in the top left panel of **(B)**] before drug, during individual and simultaneous applications of SS and Musc, and after recovery. **(A)** Right panel: difference waveforms showing changes caused by individual and simultaneous applications of SS and Musc along with a waveform resulting from summation of changes caused by individual applications of the drugs. **(B)** Left panels: amplitude by sound-pressure level (top) and amplitude by sound-frequency (bottom) functions obtained before drug, during individual and simultaneous applications of SS and Musc, and after recovery. **(B)** Right panels: changes in the peak amplitude of an LFP caused by individual and simultaneous applications of SS and Musc at various sound-pressure levels (top) and frequencies (bottom). Also, shown in each of the two panels is a curve resulting from summation of changes caused by individual applications of the drugs.

Sodium salicylate was released in combination with the GABA_A_ receptor agonist Musc in six cases, as shown by an example in Figure 10. Musc consistently suppressed the D_N_ of an LFP while SS consistently enhanced it when the drugs were released individually. During simultaneous application of the two drugs, the waveform of the D_N_ always fell between the waveforms obtained during individual applications of the drugs (e.g., Figure 10A left panel). The change caused by the drugs released simultaneously was different from the sum of the changes caused by the drugs released individually (Figure 10A right panel), suggesting that the drugs interacted with each other in regulating a response. SS-induced enhancement and Musc-induced suppression were observed over a wide range of sound intensities; and a disparity in the total effect of SS and Musc between simultaneous and individual drug applications existed over these intensities (Figure 10B).

**Figure 11:**
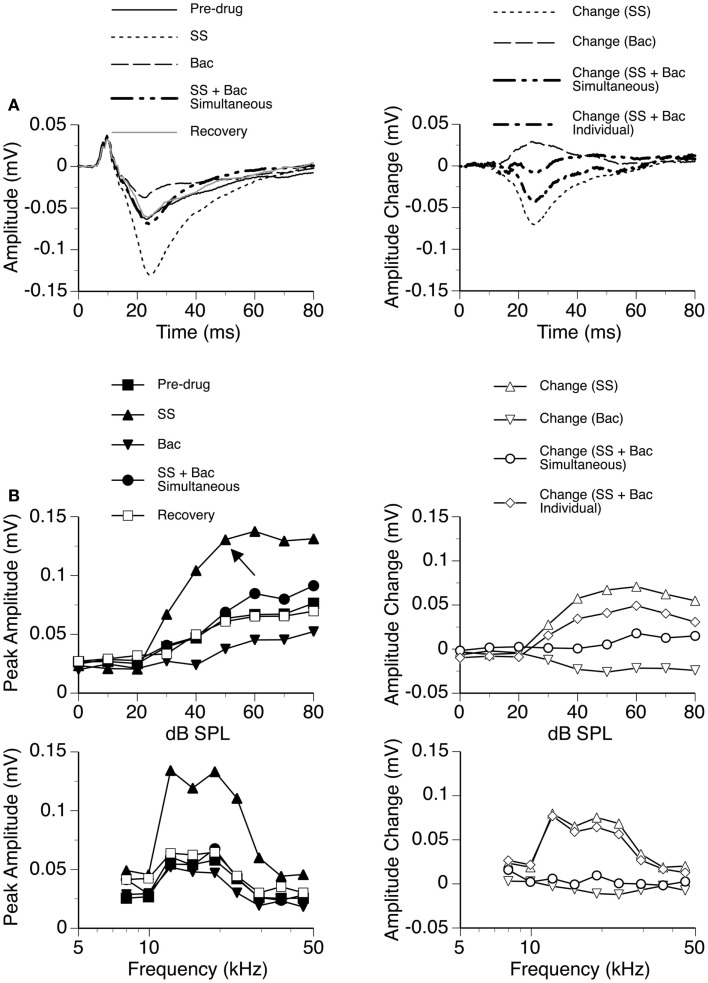
**An example showing the interaction between SS and Bac**. **(A)** Left panel: LFPs evoked by a tone burst at the BF (19.0 kHz) and 20 dB above the MT of the recording site [as indicated by an arrow in the top left panel of **(B)**] before drug, during individual and simultaneous applications of SS and Bac, and after recovery. **(A)** Right panel: difference waveforms showing changes caused by individual and simultaneous applications of SS and Bac. Also, shown in this panel is a waveform resulting from summation of changes caused by individual applications of SS and Bac. **(B)** Left panels: amplitude by sound-pressure level (top) and amplitude by sound-frequency (bottom) functions obtained before drug, during individual and simultaneous applications of SS and Bac, and after recovery. **(B)** Right panels: changes in the peak amplitude of an LFP caused by individual and simultaneous applications of SS and Bac at various sound-pressure levels (top) and frequencies (bottom). Also, shown in each of the two panels is a curve resulting from summation of changes caused by individual applications of SS and Bac.

The GABA_B_ receptor agonist Bac was released individually and in combination with SS in seven cases, as shown by an example in Figure 11. Bac consistently suppressed the D_N_ with or without the presence of SS. Difference waveforms indicated that the change caused by SS and Bac applied simultaneously was different from the sum of changes caused by the two drugs applied individually, suggesting that the drugs interacted with each other in regulating an LFP (Figures 11A,B right panels). The interaction between SS and Bac was observed over a wide range of frequencies and intensities (Figure 11B).

Results from individual cases indicated that SS and a GABAergic receptor agonist interacted with each other in regulating an LFP in the ICd. Depending on the relative strengths of the currents for releasing the drugs, the resulting waveform of D_N_ can have a positive polarity or negative polarity or a change of polarity over time. Owing to the sensitivity of the waveform to currents, no statistical analysis was performed on the interaction between SS and a receptor agonist.

## Discussion

An understanding of the effect of SS on hearing is dependent on identification of target structures of the drug and evaluation of drug-induced changes in neural activity in the structures. Furthermore, it is dependent on examination of the effect of the drug on neural mechanisms (e.g., neurotransmission) in the structures.

### Identification of ICd as a target structure of SS

Sodium salicylate reduces inputs to the central auditory system by suppressing electromotility of outer hair cells (23–26). However, systemic application of the drug does not change the amplitude of a sound-driven LFP in the IC (22, 27). This contrast suggests that the drug can directly target the IC and/or brainstem structure(s), enhancing the gain (i.e., the input–output relation) of the pathway from the cochlear nucleus to the IC (27). Such a change in neural activity offsets the reduction in the input to the central auditory system.

Using *in vitro* physiological recordings/pharmacological manipulations, people have identified the ICc and ICx as target structures of SS (34, 54). The same approach has also been used to find target structures of SS in the auditory forebrain (33, 55–57). In the *in vitro* studies, circuits associated with a neuron under investigation were only partially retained and inputs to the neuron were artificially activated. Therefore, it is difficult to use results from these studies to predict how local SS affects spontaneous and sound-driven activities of neurons in a live animal. An *in vivo* neurophysiological/pharmacological approach has been used to deal with this limitation. Using the *in vivo* approach, people have found that local SS in the AC can reduce spontaneous activity but enhance sound-driven activity in the structure (58). No results have been published about how local SS affects sound-driven activities in midbrain/brainstem structures.

In the present study, we recorded sound-evoked LFPs in the ICd while applying SS microiontophoretically at recording sites. Our results indicate that local SS can enhance auditory responses in the ICd. As SS is a small molecule that can cross blood–brain barrier, it is reasonable to believe that SS applied systemically can also reach the ICd and lead to an enhancement in auditory activity in the structure. As brain tissue is a volume conductor, an enhanced auditory response in the ICd caused by local SS can lead to an enhanced ensemble sound-driven response of the entire IC [e.g., an LFP recorded by Sun et al. (27)]. Such an enhancement can lead to an increase in the gain of the IC. It has yet to be determined whether local SS in the ICc and ICx can enhance auditory activities in the two subdivisions. Such enhancements could also lead to increases in the gain of the entire IC. A sound-driven LFP in the IC as an entity remains unchanged following systemic application of SS (27). Thus, an increase in the gain of the IC caused by local SS following systemic application of the drug should be accompanied by a reduction in the input to the structure. Further experiments are needed for evaluating the effect of SS on inputs to the IC.

An enhancement of auditory responses in the ICd by SS may cause secondary changes in the ICc and ICx through intrinsic projections (59–61). As excitatory/inhibitory natures of these projections are poorly understood, it is difficult to postulate whether auditory activities in the ICc and ICx are enhanced or suppressed. Elucidating secondary effects of SS can help fully understand the effect of the drug on the auditory midbrain.

A direct effect of SS on sound-driven LFPs in the ICd does not preclude the possibility that SS applied systemically can also enhance neural activity in the ICd through affecting sources of inputs to this structure. The ICd receives major inputs bilaterally from the AC (59, 62–67). Descending projections from the AC are likely excitatory in nature (68). An enhancement of auditory activity following systemic injection of SS has been observed in the AC (27, 28, 30, 58, 69, 70). This enhancement can very likely be reflected in sound-driven responses in the ICd. SS may also affect auditory activity in the ICd through affecting activity in the ICc and ICx, which provide intrinsic projections to the ICd (59–61). Knowledge about effects of SS on auditory responses in the ICc and ICx and information about excitatory/inhibitory natures of the intrinsic projections are needed for evaluating the dependence of auditory activity in the ICd on the ICc and ICx following systemic application of SS.

Technical limitations need to be taken into consideration in the interpretation of results from the present study. An LFP is a weighted average of potential changes generated by current dipoles at the vicinity of a recording electrode (71–73). Current dipoles located as far as a few hundred micrometers away from an electrode can contribute to an LFP (74, 75). Owing to the close proximity of the ICd to an area in the ICc where neurons with low characteristic frequencies congregate, an LFP evoked by a low-frequency sound recorded in the ICd may include contributions from these low-frequency ICc neurons. As neurons in the ICx are not tonotopically organized, a single tone burst unlikely activates many ICx neurons bordering the ICd. Therefore, contribution of the ICx to an LFP recorded in the ICd is likely limited. It is decent to believe that LFPs recorded in the present study were predominantly dependent on the ICd, in spite of possible contributions from the ICc (51).

### SS and GABAergic neurotransmission in the ICd

Our results reveal that simultaneous application of SS and a GABAergic receptor agonist or antagonist produces a change in the amplitude of an LFP different from the total change caused by the two drugs applied individually. These results suggest that SS can either directly or indirectly modulate GABAergic neurotransmission in the ICd.

Brain slice studies have revealed that SS can reduce the release of GABA in IC neurons. This reduction is at least partly due to suppression of serotonergic inputs to the GABAergic neurons (34). Such reduction can lead to disinhibition of neurons innervated by GABAergic projections. An SS-induced reduction in the release of GABA has also been observed in the AC. In this structure, SS can reduce activity of fast spiking interneurons (presumable inhibitory neurons releasing GABA) and suppress inhibitory postsynaptic currents in pyramidal neurons in layers II/III (neurons receiving GABAergic inputs) (33, 56).

Results from the present study suggest that SS can modulate GABAergic receptors. It is certain that sound-driven LFPs are different from postsynaptic potentials recorded from neurons in a brain slice preparations. However, it is believed that postsynaptic currents are among the most important current dipoles for generating an LFP (71–73, 76–78). Thus, a comparison of the time course of activation of a GABAergic receptor with the time window over which a synergistic interaction exists between SS and an antagonist of the receptor can provide useful information about the subtypes of receptors modulated by SS.

The interaction between BMI and SS was found between about 13.5 and 26 ms after the onset of a sound and was not dependent on the sound-pressure level. This time window is in general agreement with the time course of an inhibitory postsynaptic potential mediated by the GABA_A_ receptor, which peaks at about 5 ms after activation of GABAergic inputs and has a halfwidth of about 23 ms (79).

The time window over which SS had a synergistic interaction with CGP35348 was from about 20 ms to about 27.6 ms after the onset of a sound at 10 dB above the MT and was wider at high-sound-pressure levels. This fact suggests that postsynaptic GABA_B_ receptors are unlikely major targets of SS. Activation of such receptors by a single brief synaptic input can result in an inhibitory postsynaptic potential lasting for over 800 ms (80). Repetitive acoustic stimulation at a rate of 4/s (as used in the present study) would lead to sustained inhibition due to extensive temporal integration among individual postsynaptic potentials. Thus, application of SS and CGP35348 would have resulted in disinhibition over a much longer time window. Presynaptic GABA_B_ receptors regulating the release of GABA are also unlikely major targets of SS. Suppression of these receptors by SS and CGP35348 would lead to a net inhibitory effect on postsynaptic neurons and a reduction in auditory activity.

It is speculated that presynaptic GABA_B_ receptors regulating the release of glutamate may have been affected by SS. Neurotransmitter receptors activated by glutamate include the α-amino-3-hydroxy-5-methyl-4-isoxazolepropionic acid (AMPA) receptor and the *N*-methyl-d-aspartate (NMDA) receptor. A postsynaptic potential mediated by the AMPA receptor peaks at about 5 ms after the activation of a glutamatergic input and has a halfwidth <15 ms, while a potential mediated by the NMDA receptor peaks at about 25 ms and has a larger halfwidth (79). The amplitude and duration of a potential mediated by the NMDA receptor are increased by depolarization of the cell membrane. Thus, similarities exist in the time course and level dependence between SS-CGP35348 synergistic interaction and NMDA receptor-mediated postsynaptic potentials. Such similarities suggest that the enhancement in sound-driven LFP during simultaneous application of SS and CGP35348 could be due to an increase in glutamate release and consequent enhancement in postsynaptic potentials mediated by NMDA receptors. This postulation needs to be tested by future neurophysiological/pharmacological experiments.

Our results do not rule out the possibility that the effect of SS on LFPs in the ICd may also be related to direct modulation of other neurotransmitter receptors and ion channels. *In vitro* studies in the IC indicate that SS can suppress glycine receptors with α1-subunits (81). In spiral ganglion neurons, aspirin can augment responses mediated by NMDA receptors (82). In the IC and AC, SS blocks voltage-gated sodium channels and L-type calcium channels (55, 83, 84). At high doses, SS or aspirin can inhibit acid-sensing ion channels (85). All these receptors and ion channels are possible targets of SS in the ICd.

### Effects of SS: Beyond GABAergic neurotransmission in the ICd

Our results from the ICd strongly suggest that SS can enhance auditory responses through modulating local GABAergic receptors. It is likely that such modulation and a resulting enhancement in auditory response can occur beyond the ICd following systemic drug application, as GABAergic receptors also exist in other auditory structures (45–49). Enhancements of sound-driven LFPs have been found in the medial geniculate nucleus and the AC following systemic application of SS (22). Such enhancements are likely at least in part due to local modulation of GABAergic receptors.

Disinhibition in multiple structures in the auditory system may result in alterations in physiological characteristics of neural circuits. It is important to find whether reverberation of circuit and enhancement in synchronization among different auditory structures occur as a result of systemic SS application. A study of these alterations can help understand neurobiological bases of SS-induced hearing problems.

It has yet to be determined whether and how modification of GABAergic neurotransmission is responsible for SS-induced tinnitus. The relatively short-onset time of transient tinnitus (10) seems supporting that such a hearing disorder is related to direct modification of neural mechanisms. It is possible that SS-induced reduction in GABAergic inhibition not only enhances sound-driven responses (as revealed by our results) but also enables a neuron to fire action potentials in the event of receiving spontaneously generated synaptic inputs not associated with external acoustic stimuli. Such action potential firing may contribute to tinnitus.

As revealed by manganese-enhanced functional magnetic resonance imaging, expression of immediate early gene *c-fos*, and uptake of [^14^C] 2-deoxyglucose, chronic application of SS can induce tinnitus-like behavior along with a widespread increase in neural activity in the central auditory system (86–90). Structures showing a large increase in activity include the ICd. These changes caused by chronic application of SS may be in part due to direct modulation of GABAergic neurotransmission. They may also be due to alterations of other neural mechanisms, such as increase/reduction of the rate of receptor turnover and/or up/down regulation of the expression of receptor proteins. These homeostatic changes can cause side effects on hearing while helping reestablish the balance between excitation and inhibition tipped by SS.

## Conflict of Interest Statement

The authors declare that the research was conducted in the absence of any commercial or financial relationships that could be construed as a potential conflict of interest.

## Abbreviations

AC, auditory cortex; AMPA, α-amino-3-hydroxy-5-methyl-4-isoxazolepropionic acid; Bac, baclofen; BF, best frequency; BMI, bicuculline methiodide; D_N_, negative deflection of a local-field potential; D_P_, positive deflection of a local-field potential; GABA, γ-aminobutyric acid; IC, inferior colliculus; ICc, central nucleus of the inferior colliculus; ICd, dorsal cortex of the inferior colliculus; ICx, external cortex of the inferior colliculus; LFP, local-field potential; MT, minimum threshold (i.e., the threshold at the best frequency); Musc, muscimol; NMDA, *N*-methyl-d-aspartate; SS, sodium salicylate.

## References

[1] Cazals Y. Auditory sensori-neural alterations induced by salicylate. Prog Neurobiol (2000) 62:583–631. 10.1016/S0301-0082(00)00027-7 10880852

[2] Gabriel SE Fehring RA. Trends in the utilization of nonsteroidal anti-inflammatory drugs in the United States, 1986–1990. J Clin Epidemiol (1992) 45:1041–4. 10.1016/0895-4356(92)90127-9 1432021

[3] Henry JA Roberts LE Caspary DM Theodoroff SM Salvi RJ. Underlying mechanisms of tinnitus: review and clinical implications. J Am Acad Audiol (2014) 25:5–22. 10.3766/jaaa.25.1.2 24622858 PMC5063499

[4] Myers EN Bernstein JM Salicylate ototoxicity: a clinical and experimental study. Arch Otolaryngol (1965) 82:483–93 10.1001/archotol.1965.00760010485006 4954319

[5] Stolzberg D Salvi RJ Allman BL. Salicylate toxicity model of tinnitus. Front Syst Neurosci (2012) 6:28. 10.3389/fnsys.2012.00028 22557950 PMC3341117

[6] Boettcher FA Salvi RJ. Salicylate ototoxicity: review and synthesis. Am J Otolaryngol (1991) 12:33–47. 10.1016/0196-0709(91)90071-M 2029065

[7] Brien JA Ototoxicity associated with salicylates. A brief review. Drug Saf (1993) 9:143–8 10.2165/00002018-199309020-00006 8397891

[8] Grigor RR Spitz PW Furst DE. Salicylate toxicity in elderly patients with rheumatoid arthritis. J Rheumatol (1987) 14:60–6. 3572936

[9] Halla JT Hardin JG. Salicylate ototoxicity in patients with rheumatoid arthritis: a controlled study. Ann Rheum Dis (1988) 47:134–7. 10.1136/ard.47.2.134 3281604 PMC1003465

[10] Mongan E Kelly P Nies K Portor WW Paulus HE Tinnitus as an indication of therapeutic serum salicylate levels. JAMA (1973) 226:142–5 10.1001/jama.226.2.142 4740906

[11] Samlan SR Jordan MT Chan SB Wahl MS Rubin RL. Tinnitus as a measure of salicylate toxicity in the overdose setting. West J Emerg Med (2008) 9:146–9. 19561730 PMC2672263

[12] Bauer CA Brozoski TJ Rojas R Boley J Wyder M. Behavioral model of chronic tinnitus in rats. Otolaryngol Head Neck Surg (1999) 121:457–62. 10.1016/S0194-5998(99)70237-8 10504604

[13] Eggermont JJ. Hearing loss, hyperacusis, or tinnitus: what is modeled in animal research? Hear Res (2013) 295:140–9. 10.1016/j.heares.2012.01.005 22330978

[14] Jastreboff PJ Brennan JF Coleman JK Sasaki CT. Phantom auditory sensation in rats: an animal model for tinnitus. Behav Neurosci (1988) 102:811–22. 10.1037/0735-7044.102.6.811 3214530

[15] Lobarinas E Sun W Cushing R Salvi R. A novel behavioral paradigm for assessing tinnitus using schedule-induced polydipsia avoidance conditioning (SIP-AC). Hear Res (2004) 190:109–14. 10.1016/S0378-5955(04)00019-X 15051133

[16] Lobarinas E Yang G Sun W Ding D Mirza N Dalby-Brown W Salicylate- and quinine-induced tinnitus and effects of memantine. Acta Otolaryngol Suppl (2006) 556:13–9. 10.1080/03655230600895408 17114137

[17] Rüttiger L Ciuffani J Zenner HP Knipper M. A behavioral paradigm to judge acute sodium salicylate-induced sound experience in rats: a new approach for an animal model on tinnitus. Hear Res (2003) 180:39–50. 10.1016/S0378-5955(03)00075-3 12782351

[18] Turner JG. Behavioral measures of tinnitus in laboratory animals. Prog Brain Res (2007) 166:147–56. 10.1016/S0079-6123(07)66013-0 17956779

[19] Turner JG Parrish J. Gap detection methods for assessing salicylate-induced tinnitus and hyperacusis in rats. Am J Audiol (2008) 17:S185–92. 10.1044/1059-0889(2008/08-0006) 18978200

[20] Eggermont JJ Roberts LE The neuroscience of tinnitus. Trends Neurosci (2004) 27:676–82 10.1016/j.tins.2004.08.010 15474168

[21] Roberts LE Eggermont JJ Caspary DM Shore SE Melcher JR Kaltenbach JA. Ringing ears: the neuroscience of tinnitus. J Neurosci (2010) 30:14972–9. 10.1523/JNEUROSCI.4028-10.2010 21068300 PMC3073522

[22] Sheppard A Hayes SH Chen G-D Ralli M Salvi R. Review of salicylate-induced hearing loss, neurotoxicity, tinnitus and neuropathophysiology. Acta Otorhinolarylgol Ital (2014) 34:79–93. 24843217 PMC4025186

[23] Liberman MC Gao J He DZ Wu X Jia S Zuo J. Prestin is required for electromotility of the outer hair cell and for the cochlear amplifier. Nature (2002) 419:300–4. 10.1038/nature01059 12239568

[24] Kakehata S Santos-Sacchi J. Effects of salicylate and lanthanides on outer hair cell motility and associated gating charge. J Neurosci (1996) 16:4881–9. 8756420 10.1523/JNEUROSCI.16-16-04881.1996 PMC6579298

[25] Müller M Klinke R Arnold W Oestreicher E. Auditory nerve fibre responses to salicylate revisited. Hear Res (2003) 183:37–43. 10.1016/S0378-5955(03)00217-X 13679136

[26] Zheng J Shen W He DZ Long KB Madison LD Dallos P Prestin is the motor protein of cochlear outer hair cells. Nature (2000) 405:149–55 10.1038/35012009 10821263

[27] Sun W Lu J Stolzberg D Gray L Deng A Lobarinas E Salicylate increases the gain of the central auditory system. Neuroscience (2009) 159:325–34. 10.1016/j.neuroscience.2008.12.024 19154777 PMC2759817

[28] Yang G Lobarinas E Zhang L Turner J Stolzberg D Salvi R Salicylate induced tinnitus: behavioral measures and neural activity in auditory cortex of awaken rats. Hear Res (2007) 226:244–53. 10.1016/j.heares.2006.06.013 16904853

[29] Chen GD Jastreboff PJ. Salicylate-induced abnormal activity in the inferior colliculus of rats. Hear Res (1995) 82:158–78. 10.1016/0378-5955(94)00174-O 7775282

[30] Eggermont JJ Kenmochi M. Salicylate and quinine selectively increase spontaneous firing rates in secondary auditory cortex. Hear Res (1998) 117:149–60. 10.1016/S0378-5955(98)00008-2 9557985

[31] Jastreboff PJ Sasaki CT. Salicylate-induced changes in spontaneous activity of single units in the inferior colliculus of the guinea pig. J Acoust Soc Am (1986) 80:1384–91. 10.1121/1.394391 3782617

[32] Gong N Zhang M Zhang XB Chen L Sun GC Xu TL. The aspirin metabolite salicylate enhances neuronal excitation in rat hippocampal CA1 area through reducing GABAergic inhibition. Neuropharmacology (2008) 54:454–63. 10.1016/j.neuropharm.2007.10.017 18078964

[33] Wang HT Luo B Zhou KQ Xu TL Chen L. Sodium salicylate reduces inhibitory postsynaptic currents in neurons of rat auditory cortex. Hear Res (2006) 215:77–83. 10.1016/j.heares.2006.03.004 16632286

[34] Wang HT Luo B Huang YN Zhou KQ Chen L. Sodium salicylate suppresses serotonin-induced enhancement of GABAergic spontaneous inhibitory postsynaptic currents in rat inferior colliculus *in vitro*. Hear Res (2008) 236:42–51. 10.1016/j.heares.2007.11.015 18222054

[35] Xu H Gong N Chen L Xu TL. Sodium salicylate reduces gamma aminobutyric acid-induced current in rat spinal dorsal horn neurons. Neuroreport (2005) 16:813–6. 10.1097/00001756-200505310-00007 15891576

[36] Faingold CL Gehlbach G Caspary DM. On the role of GABA as an inhibitory neurotransmitter in inferior colliculus neurons – iontophoretic studies. Brain Res (1989) 500:302–12. 10.1016/0006-8993(89)90326-0 2605499

[37] Fussesery ZM Hall JC. Role of GABA in shaping frequency tuning and creating FM sweep selectivity in the inferior colliculus. J Neurophysiol (1996) 76:1059–73. 8871220 10.1152/jn.1996.76.2.1059

[38] Zhang H Kelly JB. Glutamatergic and GABAergic regulation of neural responses in inferior colliculus to amplitude-modulated sounds. J Neurophysiol (2003) 90:477–90. 10.1152/jn.01084.2002 12660357

[39] Zhang H Xu J Feng AS. Effects of GABA-mediated inhibition on direction-dependent frequency tuning in the frog inferior colliculus. J Comp Physiol A (1999) 184:86–98. 10.1007/s003590050308 10077865

[40] Ma WL Hidaka H May BJ. Spontaneous activity in the inferior colliculus of CBA/J mice after manipulations that induce tinnitus. Hear Res (2006) 212:9–21. 10.1016/j.heares.2005.10.003 16307852

[41] Adams JC Wenthold RJ Distribution of putative amino acid transmitters, choline acetyltransferase and glutamate decarboxylase in the inferior colliculus. Neuroscience (1979) 4:947–51 10.1016/0306-4522(79)90067-8 231219

[42] Kelly JB Caspary DM Pharmacology of the inferior colliculus. In: Winer JA Schreiner CE, editors. The Inferior Colliculus. New York, NY: Springer (2005). p. 248–81.

[43] Merchán M Aguilar LA Lopez-Poveda EA Malmierca MS. The inferior colliculus of the rat: quantitative immunocytochemical study of GABA and glycine. Neuroscience (2005) 136:907–25. 10.1016/j.neuroscience.2004.12.030 16344160

[44] Vater M Kössl M Horn AK. GAD- and GABA-immunoreactivity in the ascending auditory pathway of horseshoe and mustached bats. J Comp Neurol (1992) 325:183–206. 10.1002/cne.903250205 1460113

[45] Bowery NG Hudson AL Price GW. GABA_A_ and GABA_B_ receptor site distribution in the rat central nervous system. Neuroscience (1987) 20:365–83. 10.1016/0306-4522(87)90098-4 3035421

[46] Fubara BM Casseday JH Covey E Schwartz-Bloom RD. Distribution of GABA_A_, GABA_B_, and glycine receptors in the central auditory system of the big brown bat, *Eptesicus fuscus*. J Comp Neurol (1996) 369:83–92. 10.1002/(SICI)1096-9861(19960520)369:1<83::AID-CNE6>3.0.CO;2-G 8723704

[47] Glendening KK Baker BN. Neuroanatomical distribution of receptors for three potential inhibitory neurotransmitters in the brainstem auditory nuclei of the cat. J Comp Neurol (1988) 275:288–308. 10.1002/cne.902750210 2851616

[48] Jamal L Zhang H Finlayson PG Porter LA Zhang H. The level and distribution of the GABA_B_R2 receptor subunit in the rat’s central auditory system. Neuroscience (2011) 181:243–56. 10.3389/fncir.2012.00092 21371537

[49] Jamal L Khan AN Butt S Patel CR Zhang H. The level and distribution of the GABA_B_R1 and GABA_B_R2 receptor subunit in the rat’s inferior colliculus. Front Neurosci (2012) 6:92. 10.3389/fncir.2012.00092 23189044 PMC3506002

[50] Milbrandt JC Albin RL Caspary DM. Age-related decrease in GABA_B_ receptor binding in the Fischer 344 rat inferior colliculus. Neurobiol Aging (1994) 15:699–703. 10.1016/0197-4580(94)90051-5 7891824

[51] Patel CR Redhead C Cervi AL Zhang H. Neural sensitivity to novel sounds in the rat’s dorsal cortex of the inferior colliculus as revealed by evoked local field potentials. Hear Res (2012) 286:41–54. 10.1016/j.heares.2012.02.007 22406035

[52] Lumani A Zhang H. Responses of neurons in the rat’s dorsal cortex of the inferior colliculus to monaural tone bursts. Brain Res (2010) 1351:115–29. 10.1016/j.brainres.2010.06.066 20615398

[53] Slinker BK. The statistics of synergism. J Mol Cell Cardiol (1998) 30:723–31. 10.1006/jmcc.1998.0655 9602421

[54] Basta D Ernst A. Effects of salicylate on spontaneous activity in inferior colliculus brain slices. Neurosci Res (2004) 50:237–43. 10.1016/j.neures.2004.07.003 15380332

[55] Liu Y Zhang H Li X Wang Y Lu H Qi X Inhibition of voltage-gated channel currents in rat auditory cortex neurons by salicylate. Neuropharmacology (2007) 53:870–80. 10.1016/j.neuropharm.2007.08.015 17920083

[56] Su Y-Y Luo B Wang HT Chen L. Differential effects of sodium salicylate on current-evoked firing of pyramidal neurons and fast-spiking interneurons in slices of rat auditory cortex. Hear Res (2009) 253:60–6. 10.1016/j.heares.2009.03.007 19306920

[57] Su Y-Y Luo B Jin Y Wu SH Lobarinas E Salvi RJ Altered neuronal intrinsic properties and reduced synaptic transmission of the rat’s medial geniculate body in salicylate-induced tinnitus. PLoS One (2012) 7(10):e46969. 10.1371/journal.pone.0046969 23071681 PMC3468622

[58] Lu J Lobarinas E Deng A Goodey R Stolzberg D Salvi RJ GABAergic neural activity involved in salicylate-induced auditory cortex gain enhancement. Neuroscience (2011) 189:187–98. 10.1016/j.neuroscience.2011.04.073 21664433 PMC3153886

[59] Coleman JR Clerici WJ. Sources of projections to subdivisions of the inferior colliculus in the rat. J Comp Neurol (1987) 262:215–26. 10.1002/cne.902620204 3624552

[60] González-Hernández TH Meyer G Ferres-Torres R. The commissural interconnections of the inferior colliculus in the albino mouse. Brain Res (1986) 368:268–76. 10.1016/0006-8993(86)90571-8 2421840

[61] Malmierca MS Hernandez O Falconi A Merchan M Rees A The commissure of the inferior colliuclus, anatomical and physiological correlates. Assoc Res Otolaryngol Abstr (2001) 24:191.

[62] Bajo VM Nodal FR Bizley JK Moore DR King AJ. The ferret auditory cortex: descending projections to the inferior colliculus. Cereb Cortex (2007) 17:475–91. 10.1093/cercor/bhj164 16581982 PMC7116556

[63] Druga R Syka J Rajkowska G. Projections of auditory cortex onto the inferior colliculus in the rat. Physiol Res (1997) 46:215–22. 9728510

[64] Herbert H Aschoff A Ostwald J Topography of projections from the auditory cortex to the inferior colliculus in the rat. J Comp Neurol (1991) 304:103–22 10.1002/cne.903040108 2016407

[65] Herrera M Hurtado-García JF Collia F Lanciego J. Projections from the primary auditory cortex onto the dorsal cortex of the inferior colliculus in albino rats. Arch Ital Biol (1994) 132:147–64. 7526815

[66] Malmierca MS Ryugo DK Descending connections of auditory cortex to the midbrain and brain stem. In: Winer JA Schreiner CE, editors. The Auditory Cortex. New York, NY: Springer (2010). p. 189–208.

[67] Saldaña E Feliciano M Mugnaini E. Distribution of descending projections from primary auditory neocortex to inferior colliculus mimics the topography of intracollicular projections. J Comp Neurol (1996) 371:15–40. 10.1002/(SICI)1096-9861(19960715)371:1<15::AID-CNE2>3.0.CO;2-O 8835717

[68] Feliciano M Potashner SJ. Evidence for a glutamatergic pathway from the guinea pig auditory cortex to the inferior colliculus. J Neurochem (1995) 65:1348–57. 10.1046/j.1471-4159.1995.65031348.x 7643112

[69] Norena AJ Moffat G Blanc JL Pezard L Cazals Y. Neural changes in the auditory cortex of awake guinea pigs after two tinnitus inducers: salicylate and acoustic trauma. Neuroscience (2010) 166:1194–209. 10.1016/j.neuroscience.2009.12.063 20096752

[70] Zhang X Yang P Cao Y Qin L Sato Y. Salicylate induced neural changes in the primary auditory cortex of awake cats. Neuroscience (2011) 172:232–45. 10.1016/j.neuroscience.2010.10.073 21044658

[71] Logothetis NK The underpinnings of the BOLD functional magnetic resonance imaging signal. J Neurosci (2003) 23:3963–71. 12764080 10.1523/JNEUROSCI.23-10-03963.2003 PMC6741096

[72] Logothetis NK. What we can do and what we cannot do with fMRI. Nature (2008) 453:869–78. 10.1038/nature06976 18548064

[73] Goense JB Logothetis NK. Neurophysiology of the BOLD fMRI signal in awake monkeys. Curr Biol (2008) 18:631–40. 10.1016/j.cub.2008.03.054 18439825

[74] Freeman WJ Mass Action in the Nervous System. New York, NY: Academic Press (1975).

[75] Legatt AD Arezzo J Vaughan HG. Averaged multiple unit activity as an estimate of phasic changes in local neuronal activity: effect of volume-conducted potentials. J Neurosci Methods (1980) 2:203–17. 10.1016/0165-0270(80)90061-8 6771471

[76] Burkard RF Secor CA Salvi RJ. Near-field responses from the round window, inferior colliculus, and auditory cortex of the unanesthetized chinchilla: manipulations of noiseburst level and rate. J Acoust Soc Am (1999) 106:304–31. 10.1121/1.427058 10420623

[77] Mitzdorf U Current source-density method and application in cat cerebral cortex: investigation of evoked potentials and EEG phenomena. Physiol Rev (1985) 65:37–100. 3880898 10.1152/physrev.1985.65.1.37

[78] Mitzdorf U. Properties of the evoked potential generators: current source-density analysis of visually evoked potentials in the cat cortex. Int J Neurosci (1987) 33:33–59. 10.3109/00207458708985928 3610492

[79] Wu SH Ma CL Kelly JB. Contribution of AMPA, NMDA, and GABAA receptors to temporal pattern of postsynaptic responses in the inferior colliculus of the rat. J Neurosci (2004) 24:4625–34. 10.1523/JNEUROSCI.0318-04.2004 15140934 PMC6729405

[80] Sun H Wu SH. The physiological role of pre- and postsynaptic GABA(B) receptors in membrane excitability and synaptic transmission of neurons in the rat’s dorsal cortex of the inferior colliculus. Neuroscience (2009) 21:198–211. 10.1016/j.neuroscience.2009.02.011 19409201

[81] Lu YG Tang ZQ Ye ZY Wang HT Huang YN Zhou KQ Salicylate, an aspirin metabolite, specifically inhibits the current mediated by glycine receptors containing alpha1-subunits. Br J Pharmacol (2009) 157:1514–22. 10.1111/j.1476-5381.2009.00321.x 19594751 PMC2765319

[82] Peng BG Chen S Lin X. Aspirin selectively augmented N-methyl-D-aspartate types of glutamate responses in cultured spiral ganglion neurons of mice. Neurosci Lett (2003) 343:21–4. 10.1016/S0304-3940(03)00296-9 12749988

[83] Liu Y Li X. Effects of salicylate on voltage-gated sodium channels in rat inferior colliculus neurons. Hear Res (2004) 193:68–74. 10.1016/j.heares.2004.03.006 15219321

[84] Liu Y Li X Ma C Liu J Lu H. Salicylate blocks L-type calcium channels in rat inferior colliculus neurons. Hear Res (2005) 205:271–6. 10.1016/j.heares.2005.03.028 15953536

[85] Wang W Ye SD Zhou KQ Wu LM Huang YN. High doses of salicylate and aspirin are inhibitory on acid-sensing ion channels and protective against acidosis-induced neuronal injury in the rat cortical neuron. J Neurosci Res (2012) 90:267–77. 10.1002/jnr.22742 21969311

[86] Holt AG Bissig D Mirza N Rajah G Berkowitz B. Evidence of key tinnitus-related brain regions documented by a unique combination of manganese-enhanced MRI and acoustic startle reflex testing. PLoS One (2010) 5:e14260. 10.1371/journal.pone.0014260 21179508 PMC3002264

[87] Wallhausser-Franke E. Salicylate evokes c-fos expression in the brain stem: implications for tinnitus. Neuroreport (1997) 8:725–8. 10.1097/00001756-199702100-00029 9106755

[88] Wallhausser-Franke E Braun S Langner G. Salicylate alters 2-DG uptake in the auditory system: a model for tinnitus? Neuroreport (1995) 7:1585–8. 10.1097/00001756-199607080-00010 8904760

[89] Wallhausser-Franke E Mahlke C Oliva R Braun S Wenz G Langner G. Expression of c-fos in auditory and non-auditory brain regions of the gerbil after manipulations that induce tinnitus. Exp Brain Res (2003) 153:649–54. 10.1007/s00221-003-1614-2 14508632

[90] Wu JL Chiu TW Poon PW. Differential changes in Fos-immunoreactivity at the auditory brainstem after chronic injections of salicylate in rats. Hear Res (2003) 176:80–93. 10.1016/S0378-5955(02)00747-5 12583883

